# Increased risk of gastric adenocarcinoma after treatment of primary gastric diffuse large B-cell lymphoma

**DOI:** 10.1186/1471-2407-13-499

**Published:** 2013-10-26

**Authors:** Koji Inaba, Ryoji Kushima, Naoya Murakami, Yuuki Kuroda, Ken Harada, Mayuka Kitaguchi, Kotaro Yoshio, Shuhei Sekii, Kana Takahashi, Madoka Morota, Hiroshi Mayahara, Yoshinori Ito, Minako Sumi, Takashi Uno, Jun Itami

**Affiliations:** 1 Department of Radiation Oncology, National Cancer Center Hospital, 5-1-1 Tsukiji, Chuo-ku, Tokyo 104-0045, Japan; 2 Department of Pathology and Clinical Laboratories, Pathology Division, National Cancer Center Hospital, 5-1-1 Tsukiji Chuo-ku, Tokyo 104-0045, Japan; 3 Diagnostic Radiology and Radiation Oncology, Graduate School of Medicine, Chiba University, 1-8-1, Inohana, Chuo-ku, Chiba City, Chiba 260-8670, Japan

**Keywords:** Gastric lymphoma, Metachronous gastric adenocarcinoma, Diffuse large B-cell lymphoma, Radiotherapy

## Abstract

**Background:**

There have been sporadic reports about synchronous as well as metachronous gastric adenocarcinoma and primary gastric lymphoma. Many reports have dealt with metachronous gastric adenocarcinoma in mucosa-associated lymphoid tissue lymphoma of stomach. But to our knowledge, there have been no reports that document the increased incidence of metachronous gastric adenocarcinoma in patients with gastric diffuse large B-cell lymphoma. This retrospective study was conducted to estimate the incidence of metachronous gastric adenocarcinoma after primary gastric lymphoma treatment, especially in diffuse large B-cell lymphoma.

**Methods:**

The retrospective cohort study of 139 primary gastric lymphoma patients treated with radiotherapy at our hospital. Mean observation period was 61.5 months (range: 3.7-124.6 months). Patients profile, characteristics of primary gastric lymphoma and metachronous gastric adenocarcinoma were retrieved from medical records. The risk of metachronous gastric adenocarcinoma was compared with the risk of gastric adenocarcinoma in Japanese population.

**Results:**

There were 10 (7.2%) metachronous gastric adenocarcinoma patients after treatment of primary gastric lymphomas. It was quite high risk compared with the risk of gastric carcinoma in Japanese population of 54.7/100,000. Seven patients of 10 were diffuse large B-cell lymphoma and other 3 patients were mixed type of diffuse large B-cell lymphoma and mucosa associated lymphoid tissue lymphoma. Four patients of 10 metachronous gastric adenocarcinomas were signet-ring cell carcinoma and two patients died of gastric adenocarcinoma. Metachronous gastric adenocarcinoma may have a more malignant potential than sporadic gastric adenocarcinoma. Old age, Helicobacter pylori infection and gastric mucosal change of chronic gastritis and intestinal metaplasia were possible risk factors for metachronous gastric adenocarcinoma.

**Conclusion:**

There was an increased risk of gastric adenocarcinoma after treatment of primary gastric lymphoma, especially of diffuse large B-cell lymphoma.

## Background

There have been sporadic reports about synchronous as well as metachronous gastric adenocarcinoma (GA) and primary gastric lymphoma (PGL) [1-16]. Regarding synchronous GA and PGL, Ishihama et al. reported that GA was seen synchronously in 3.3% of PGL patients (4/121 patients) [8]. Concerning the metachronous GA and PGL, the largest study is that of Capelle LG et al. which reported that metachronous GA risk was six times higher in the patients with gastric mucosa associated lymphoid tissue (MALT) lymphoma than in the Dutch general population [16]. Many reports have dealt with metachronous GA in MALT lymphoma of stomach. But to our knowledge, there have been no reports that document the increased incidence of metachronous GA in patients with gastric diffuse large B-cell lymphoma (DLBCL). This retrospective study was conducted to estimate the incidence of metachronous GA after PGL treatment, especially in DLBCL.

## Methods

The radiation oncology department database from 2000 to 2010 was searched for PGL patients who underwent radiotherapy. Patient’s profiles at the time of PGL diagnosis (age, sex, lymphoma histology, stage, treatment and outcome) were retrieved retrospectively. After treatment of PGL, patients underwent regular follow-ups including endoscopic examinations and CT scannings. The date of GA diagnosis, interval between PGL and GA, stage of GA, treatment of GA and outcome were also investigated. One of the authors, an experienced gastrointestinal pathologist, reviewed the biopsy slides at the time of PGL and GA diagnosis and the presence of atrophic gastritis and intestinal metaplasia was diagnosed. In the 10 patients with metachronous GA after PGL, the presence of Helicobacter pylori (HP) infection was diagnosed retrospectively by pathology findings of the biopsy specimens or by results of the various laboratory tests.

Estimated risk of GA after PGL was calculated in comparison to the incidence of GA in Japanese population. Our institutional review board (National Cancer Center Institutional Review Board) approved this study.

## Results

**Figure 1:**
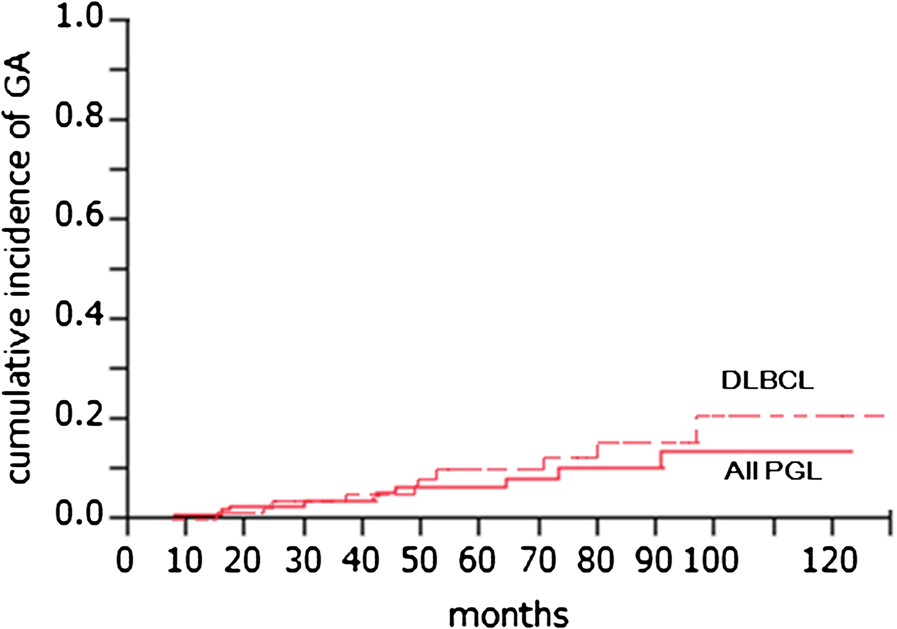
**Incidence of GA/ all PGL and DLBCL.** GA: gastric adenocarcinoma, PGL: primary gastric lymphoma, DLBCL: diffuse large B-cell lymphoma.

There were 139 patients with PGL (MALT lymphoma in 51 patients, DLBCL in 83, peripheral T-cell lymphoma (PTCL) in one, adult T-cell lymphoma (ATL) in 2 and follicular lymphoma (FL) in 2). Eighteen patients with MALT lymphoma were treated by radiation therapy because of the residual tumors after HP eradication, and the remaining 33 patients underwent radiation therapy because they were HP negative. Patient characteristics are shown in Table 1. Mean observation length was 61.5 months (range: 3.7-124.6 months). All PGLs in the current study were treated by radiotherapy and almost all patients except MALT were treated with chemo-radiotherapy. There were seen 10 patients with GA developed after treatment of PGL. No synchronous GA was found in this study. Tables 2 and 3 show the details of metachronous GA. The mean latent period between PGL and GA was 43.1 months (range: 7.9-90.8 months) (Figure 1). The histological types of GA were well differentiated adenocarcinoma in 4 patients, well to moderate differentiated adenocarcinoma in one, well to poorly differentiated adenocarcinoma in one, and signet-ring cell carcinoma in 4. In 9 patients, GA was classified as stage I and the remaining one was diagnosed as stage II. About the lymphoma histology, seven patients were classified as DLBCL and the remaining 3 showed a mixture of DLBCL and MALT lymphoma. For the GA, endoscopic resection was performed in 6 patients with no relapses. Four patients underwent surgery with relapses seen in 2 patients. Both of them succumbed to distant metastasis 9 and 37 months after surgery despite chemotherapy. Two- and 5-year overall survivals of GA were 90.0% and 75.0%. The 5-year overall survivals of DLBCL and MALT lymphoma were 89.6% and 97.7% respectively. Eight of the 83 DLBCL patients died, of which 5 were from non-lymphoma causes. Two of the 5 died from metachronous GA.

**Table 1:** Patient’s characteristic and PGL treatment

			**DLBCL**		**MALT lymphoma**		**Others**	
**N**	**139**		**83**		**51**		**5 (ATL 2, FL 2, PTCL 1)**	
**Age**	**Median 62 ( range: 19–85)**		**Median 63 ( range: 20–85)**		**Median 56 ( range: 19–76)**		**Median 64 ( range: 53–72)**	
Sex (M : F)	71 : 68		44 : 39		25 : 26		2 : 3	
Stage	I	94	I	42	I	48	I	4
(Lugano)	II	40	II	37	II	2	II	1
	IV	5	IV	4	IV	1		
Treatment	RT	50	RT	0	RT	49	RT	1
	CT+RT	89	CT+RT	83	CT+RT	2	CT+RT	4
Rituximab	R+	40	R+	38	R+	1	R+	1
	R-	99	R-	45	R-	50	R-	4
RT dose			40 Gy/20fr	35	30 Gy/15fr	23	40 Gy/20fr	2
	40 Gy≧	89	40.5 Gy/27fr	45	30 Gy/20fr	24	40.5 Gy/27fr	2
	40 Gy<	50	44 Gy/22fr	2	36 Gy/18fr	1	36 Gy/18fr	1
			30 Gy/15fr	1	40 Gy/20fr	1		
					46 Gy/23fr	2		
Observation length	mean 61.5 months		mean 62.7 months		mean 61.0 months		mean 46.9 months	
(range)	(3.7–124.6)		(7.1–124.6)		(3.7–120.4)		(11.5–82.4)	
5 year OS	92.0%		89.6%		97.7%		75.0%	

PGL: primary gastric lymphoma.

DLBCL: diffuse large B-cell lymphoma.

MALT: mucosa-associated lymphoid tissue.

ATL: adult T-cell lymphoma.

FL: follicular lymphoma.

PTCL: peripheral T-cell lymphoma.

RT: radiotherapy.

CT: chemotherapy.

OS: overall survival.

R: rituximab.

**Table 2:** Metachronous GA and details

**No**	**Age**	**Sex**	**PGL pathology**	**Lugano stage**	**Tx for PGL**	**Interval (months) from PGL to GA**	**GA pathology**	**Stage for GA**	**Tx for GA**	**Outcome**
1	72	F	DLBCL	II1	CHOP×3	42.6	sig	m	distal gastrectomy	Alive for 3.7 m after GA
					+					
					40 Gy/20fr					
2	62	M	DLBCL	I	CHOP×3	64.3	W/D AC	m	ESD	Alive for 47.7 m after GA
					+					
					40.5 Gy/27fr					
3	70	M	DLBCL	II1	CHOP×3	45.9	W to M/D AC	m	ESD	Alive for 71.6 m after GA
					+					
					40.5 Gy/27fr					
4	68	F	DLBCL	I	CHOP×3	90.8	W/D AC	m	ESD	Alive for 31.8 m after GA
					+					
					40.5 Gy/27fr					
5	70	M	DLBCL	II1	CHOP×3	16.0	W/D AC	sm	EMR→partial gastrectomy	Dead for 36.9 m after GA
					+					
					40.5 Gy/27fr					
6	63	M	DLBCL	IV	R×8-CHOP×8	30.2	W to P/D AC	se	total gastrectomy	Dead for 12.0 m after GA
					+					
					40.5 Gy/27fr					
7	36	M	MALT	II1	R×8-CHOP×3	42.1	sig	m	partial gastrectomy	Alive for 20.6 m after GA
			+		+					
			DLBCL		40 G y/20fr					
8	63	M	DLBCL	II1	R×8-CHOP×6	73.7	sig	m	EMR	Alive for 16.6 m after GA
					+					
					40.5 Gy/27fr					
9	66	F	MALT	II1	CHOP×3	17.5	sig	m	EMR	Alive for 69.3 m after GA
			+		+					
			DLBCL		40.5 Gy/27fr					
10	73	F	MALT	I	CHOP×4	7.9	W/D AC	m	EMR	Alive for 70.2 m after GA
			+		+					
			DLBCL		40.5 Gy/27fr					

sig: signet-ring cell carcinoma.

W/D: well differentiated.

M/D: moderately differentiated.

P/D: poorly differentiated.

AC: adenocarcinoma.

PGL: primary gastric lymphoma.

R: rituximab.

Tx: treatment.

GA: gastric adenocarcinoma.

CHOP: cyclophosphamide, hydroxydaunorubicin, vincristine and prednisolone.

**Table 3:** Metachronous GA and pathologic change

**No**	**Gastric mucosa at PGL**	**Intestinal metaplasia at PGL**	**Gastric mucosa at GA**	**Intestinal metaplasia at GA**	**HP infection**
1	atorophic	+	atorophic	+	possible
2	atorophic	+	atorophic	+	+
3	atorophic	+	atorophic	+	possible
4	atorophic	+	atorophic	+	+
5	atorophic	-	atorophic	+	+
6	atorophic	-	atorophic	+	possible
7	normal	-	atorophic	+	+
8	atorophic	-	atorophic	+	+
9	atorophic	+	atorophic	+	possible
10	atorophic	+	atorophic	+	possible

HP infection possible means background gastric mucosal changes suggesting HP infection.

In the 10 patients with metachronous GA after PGL, mucosal findings at the time of PGL showed atrophic gastritis in 9 patients and intestinal metaplasia in 6. In contrast, the mucosa at the time of GA diagnosis showed atrophic gastritis and intestinal metaplasia in all 10 patients. Of 10 patients who developed GA after PGL, 5 had HP infection, and the remaining 5 patients had no information of HP infection but the background gastric mucosal changes of atrophic gastritis and intestinal metaplasia suggested possible presence of HP infection (Table 3).

Table 4 compared factors between the whole patients and the patients with metachronous GA. The incidence of metachronous GA was higher in the patients with age over 60 years, DLBCL, presence of HP infection, chemotherapy and stage II-IV with a statistical significance and the presence of intestinal metaplasia of gastric mucosa with a marginal significance (p=0.07).

**Table 4:** The comparison with metachronous GA and without metachronous GA

	**n**	**Metachronous GA+**	**p value**	**Odds ratio**	**95% CI**
Sex					
M	71	6 (8%)	0.4	1.47	0.40–5.48
F	68	4 (6%)			
Age					
60≦	64	1 (2%)	0.02^※^	8.59	1.06–69.78
60>	75	9 (12%)			
Histology of Lymphoma					
DLBCL	83	10 (12%)	0.007^※^	-	-
MALT	51	0			
HP Infection					
HP positive	22	5 (23%)	0.007^※^	-	-
HP negative	34	0			
Chemotherapy					
Yes	88	10 (11%)	0.009^※^	-	-
No	51	0			
Rituximab					
Yes	40	3 (8%)	0.59	1.07	0.26–4.34
No	99	7 (7%)			
Stage					
Stage I	93	3 (3%)	0.02^※^	5.38	1.32–21.92
Stage II-IV	46	7 (15%)			
Chronic Gastritis					
Yes	65	9 (14%)	0.26	3.05	0.36–25.71
No	20	1 (5%)			
Intestinal Metaplasia					
Yes	29	6 (21%)	0.07	3.39	0.87–13.17
No	56	4 (7%)			

GA: gastric adenocarcinoma.

CI: confidence interval.

※ p<0.05.

The risk of metachronous GA after PGL was 10/139(=7.2%). If confined to DLBCL, the risk was 10/83(=12.0%) (Figure 1). The risk of GA in Japanese population is about 54.7/100,000 [17]. The relative risk was 131 (95% confidence interval: 68–251). If confined to DLBCL, the relative risk was 219 (95% confidence interval: 116–415).

## Discussion

There were 10 patients developing metachronous GA after treatment of PGL. The risk of metachronous GA was higher in the patients with age over 60 years, DLBCL, HP infection, use of chemotherapy, Stage II-IV of PGL. In this study, MALT lymphomas were treated by radiation therapy after HP eradication as described in European Gastro-Intestinal Lymphoma Study (EGILS) group consensus report [18]. HP eradication and HP negative may explain that MALT lymphomas in this study showed no increased risk of metachronous GA. Increased risk of GA in Stage II-IV and patients treated with chemotherapy can be explained by the fact that only the patients with DLBCL showed advanced stages and treated by chemotherapy. Multivariate analysis to estimate factors for metachronous GA showed no statistical significance due to small number.

HP infection is well known in causing atrophic gastritis and intestinal metaplasia which might lead to GA. Not only the ongoing HP infection, but also radiation and chemotherapy could have contributed the mucosal change.

Old patients with primary gastric diffuse large B-cell lymphoma (PGDLBCL) who remains HP-infection positive and have mucosal change of atrophic gastritis and intestinal metaplasia should be followed up cautiously early to find metachronous GA and eradication of HP infection should be considered.

The increased risk of GA after PGL might be possibly explained by 2 theories. One is that damages to gastric mucosa by HP infection, chemotherapy, or radiotherapy increase GA risk [19-21], and the other is that GA remained undetected at the time of PGL because GA was so small [22-24]. Risk of treatment-related solid cancer increases more than 5 years after the primary treatment. The interval of GA and PGL was within 5 years in 7 patients. These patients with short interval may have undetected small GA at the time of PGL and the patients with longer interval could be caused by continuing HP infection and/or treatment.

To our knowledge, this is the first report which has large number of PGL and longtime follow-up and shows the high risk of GA after treatment of PGL, especially in DLBCL.

Regarding the nature of GA after PGL, cancerous infiltration was only up to mucosal layer in 8 patients. This early detection was due to the short intervals between endoscopic examinations for PGL follow-ups. However, 4 of 10 patients had signet-ring cell carcinomas that had a more aggressive behavior than differentiated adenocarcinoma. Additionally, 2 patients with submucal and serosal infiltrations succumbed to distant metastasis after gastrectomy with 2 year and 5 year overall survival of 90.0% and 75.0%, respectively. GA after PGL may have more malignant potential than sporadic GA.

## Conclusion

There were 10 patients of metachronous GA after PGL and the risk of metachronous GA after PGDLBCL was 10/83(=12.0%). GA after PGL may have more malignant potential than sporadic GA. Eradication of HP infection should be considered in PGL to reduce the risk of GA.

## Competing interests

The authors declare that they have no competing interests.

## Authors’ contributions

KI, YK, MM, HM, YI, MS, TU and JI have made substantial contributions to conception and design. KI, TU and JI have been involved in drafting the manuscript or revising it critically for important intellectual content. RK carried out the pathologic examination. KI, NM, KH, MK, KY, SS and KT participated in acquisition of data and interpretation of data. All authors read and approved the final manuscript.

## Pre-publication history

The pre-publication history for this paper can be accessed here:

http://www.biomedcentral.com/1471-2407/13/499/prepub
